# Influence of UV Ageing on Properties of Printed PLA Containing Graphene Nanopowder

**DOI:** 10.3390/ma15228135

**Published:** 2022-11-16

**Authors:** Leszek Czechowski, Slawomir Kedziora, Elvin Museyibov, Markus Schlienz, Piotr Szatkowski, Martyna Szatkowska, Jacek Gralewski

**Affiliations:** 1 Department of Strength of Materials, Lodz University of Technology, 90-537 Lodz, Poland; 2 Faculty of Science, Technology and Medicine, Luxembourg University, L-1359 Luxembourg, Luxembourg; 3 Faculty of Materials Science and Ceramics, University of Science and Technology, 30-059 Krakow, Poland; 4 Institute of Marketing and Sustainable Development, Lodz University of Technology, 93-590 Lodz, Poland

**Keywords:** graphene nanopowder, mechanical properties, composite material, polylactic acid, 3-dimensional printing

## Abstract

The present paper analyses the properties of printed polylactic acid (PLA) samples with admixtures of graphene nanopowder (GNP) at wt. 1%, 2% and 4%. The pure polylactide and admixed polylactide printed samples were examined to determine their chemical-physical properties, stiffness, and strength parameters. The tests of tensile, dynamic mechanical analysis (DMA), difference thermogravimetric (TG), and differential scanning calorimetry (DSC) were executed before and after UV (ultraviolet) treatment. The first part of the paper shows the process of manufacturing granulates and filaments mixed with graphene. The second part of the paper concerns the results of the tests made on printed samples. The analysed samples were printed using a Prusa i3 MK3 printer. It transpired that the content of graphene at 1% improved the mechanical parameters of the printed composite by organising its structure. Increasing the amount of graphene caused the values of the measured parameters to drop. This research indicates how important it is to determine the optimal values of nanoadditives in biopolymers.

## 1. Introduction

The industry of manufacturing printed materials, commonly called 3D printing or additive manufacturing (AM), is being strongly developed in many branches. 3D printing technology, by reason of low costs and flexibility, can be applied in prototyping phases. The simplest materials taken into consideration are various polymers. The most-used method of 3D printing is fused deposition modelling (FDM), which relies upon forming a printed shape by using melted wire (filament) under certain physical conditions. Currently, PLA filament is often used in 3D printing because this material is characterised not only by good thermo-plasticity and moderately high mechanical properties, but also by good biodegradability and compatibility. PLA, regarded as biodegradable biopolymers, can be produced from plants, bacteria, mushrooms, or crustacean shells. To enhance the general strength and stiffness of polymers, some reinforcements are applied and added into the structure of polymers. These reinforcements are mostly additives such as glass, graphite, carbon, or organic fibres. Therefore, many recent works have explored the influence of different reinforcements in polymers, especially on their mechanical properties. Sanes et al. [1], analysed thermoplastic nanocomposite materials with different forms of graphene or graphene oxide nanofillers. Dou et al. [2], verified the mechanical properties of carbon fibre-reinforced PLA samples produced by 3D printing. They indicated that the relative content of fibre can significantly influence a sample’s mechanical properties. Figueroa-Velarde et al. [3], examined PLA samples filled with agave fibres. The analysed composite material was obtained by Fused Deposition Modelling (FDM)—a 3D printing technique. Prajapati et al. [4], investigated fibre-reinforced polymer composites (FRPC) to determine their impact strength. Kuschmitz et al. [5] explored the characterisation of printed samples based on carbon and flax fibres. The authors of paper [6] presented the analysis of an biocompatible graft copolymer (3,4-ethylenedioxythiophene) manufactured by the 3D printing melting extrusion method. They stated that the copolymer is characterised by excellent cell growth and maturation of neonatal cardiac myocytes. Paper [7] concerns the analysis of the properties of 3D-printed PLA blended with carbon fibre (CF) and graphene oxide (GO) by applying polybutylene succinate. The authors revealed that compared to pure PLA, the tensile strength was 73.33% higher, and the tensile modulus was 231.71% greater. Liu et al. [8], analysed the thermal conductivity of PLA printed samples filled with SiC and carbon obtained using FDM. Grant et al. [9], considered the influence of print orientation on mechanical properties based on PLA and ABS samples. They stated that statically, the higher properties (stress and strain) are for orientation 0°. The study confirming the influence of different plasticisers, such as epoxidised soybean oil (ESO), polyethylene glycol (PEG), and glycerol, on the mechanical and thermal properties of printed PLA samples was included in work [10]. It was shown in paper [11] that the mechanical performance of the continuous carbon fibre-reinforced PLA samples printed in a vacuum can be higher than the same samples printed in the atmospheric environment. PLA scaffold structure with copper, bronze, and silver particle additives can be used in biomedicine [12]. However, applying some reinforcements to the samples does not always enhance the mechanical properties sufficiently [13,14,15,16]. Tang et al. [17], analysed the mechanical properties of printed PLA samples and lattice structures. Various modifiers applied in polylactide can affect the duration of biodegradation [18]. The investigation into physical ageing, morphology, and mechanical properties provides some techniques to improve the strength of PLA-based materials [19,20]. The literature contains many studies on PLA composites modified with carbon nanoforms [21,22]. In most applications, nanographene is used as a functional modifier, e.g., for improving thermal conductivity [23].

Based on the above-mentioned literature, there is a lot of research on graphene-modified polymers, but there are few analyses that concern the influence of the addition of significant amounts of graphene used in PLA samples, especially those printed from own prepared filament; therefore, this work provides a new look at these and their results. Previously, we performed similar research for samples of small additives of graphene [24], in which we discussed the influence of graphene on PLA. In this analysis, PLA/graphene composites produced by a completely different technology (10 kN piston injection) were tested. In comparing the results obtained by us in the previous article to the present one, some similarities can be noticed, primarily the increase in strength after ageing in UV and moist soil. It was stated that the mechanisms responsible for this were primarily the relaxation of shorter chains after the degradation process. The quality of the produced biocomposites based on PLA with the graphene modifier is influenced not only by the quality of the starting materials, but also by the technology of their production. PLA and/or PLA reinforced with graphene (by applying 3D-printed materials) have recently been analysed in the literature, with the study of their respective tensile properties, e.g., the paper of Caminero et al. [25], in which the properties of natural and modified PLA, or PLA-graphene, were studied; in this work, both the first and the second filaments applied in the preparations of 3D printed samples were manufactured by external companies. In addition, the authors did not give a clear content of graphene used in PLA. They performed tensile, three-point bending, Charpy impact, and interlaminar shear strength tests to determine the mechanical response of the 3D printed unaged specimens. Another paper [26] deals with an analysis of only the tensile test of PLA unaged samples reinforced with addition of 1% and 2% graphene nanoplatelets printed from filament fabricated by the Directa Plus company. The authors of this article concluded that the presence of graphene in PLA samples does not increase the strength of the material. Moreover, determinations of Young’s moduli were not taken into consideration. In contrast to the aforementioned papers, in the present work we verified not only the essential parameters and the influence of graphene on such a printed element before and after the ageing process, but also the dependence of the presence of graphene on the loss of these properties over time. The filament of pure PLA needed to print the samples was produced using PLA pellets and a Filabot EX2 extruder. The elaborated filament is a mixture of PLA and graphene nanopowder and was produced from delivered pellets and according to our own procedure. This enabled producing the filament with quite a great content of graphene. The analysis was expanded to explore biodegradable PLA printed samples admixed with GNP at wt. 1%, 2% and 4%. The application of great contents of nanographene in printed samples required appropriate preparation of the filament and/or adequate setting of the printing, as was performed here. The one-directional tensile tests were performed on samples to determine their mechanical properties. All the tensile curves have been presented, and characteristic parameters have been sorted into the appropriate tables. Moreover, tests of thermogravimetric (TG), dynamic mechanical analysis (DMA), and differential scanning calorimetry (DSC) were also conducted on samples before and after UV ageing. The results of the present work could be profitable in the case of an application of graphene in PLA, in order to control the material properties due to the ageing effect.

## 2. Methodology

### 2.1. Sample Manufacturing Processing

**Figure 1:**
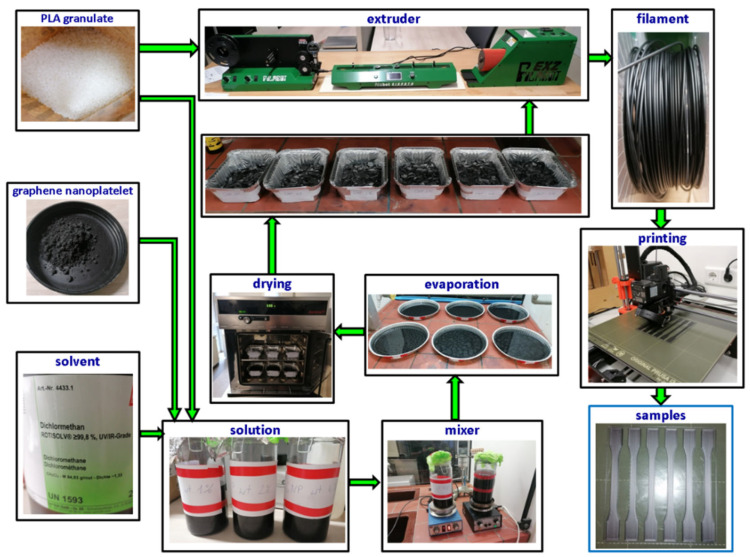
Schematic course of preparing printed samples made of pure PLA and PLA with GNP.

The first step of preparation of the samples was based on manufacturing the filament from PLA pellets (fully stabilized, Smartmaterials3d, Jaén, Spain) and graphene nanoplatelets (delivered by Nanografi company, purity 99.9%+, size 5 nm, diameter 7 µm). The melting temperature in the case of pure PLA and PLA admixed with graphene nanopowder (GNP) was set in the range of 180–185 °C and 150–170 °C, respectively. Setting the parameters of manufacturing pure PLA filament allowed us to achieve PLA wire with the correct diameter of 1.7–1.8 mm. Achieving the correct diameter was essential to create adequate samples during printing. An effective technique for manufacturing the PLA filament was associated with a cooling system which enabled a very fast decrease in the temperature of the hot PLA wire coming from the extruder nozzle. In the case of PLA filament with additives of GNP, the batch to extruder was adequately prepared. The schematic illustration of admixing the batch composed of PLA and graphene pellets is displayed in Figure 1. At the beginning, PLA granulate and graphene pellets measured out in adequate proportion (wt. 1%, 2%, 4% GNP) were inserted into jars filled with solvent dichloromethane. The closed jars with solutions were put aside for 24 h. Subsequently, the process involved mixing the solutions for a few hours. Afterwards, evaporation followed for 24 h. Chopped pieces of PLA and graphene were dried in the oven for the next 24 h at a temperature of 65–70 °C. The very low moisture of PLA-graphene composite would not necessarily have been achieved otherwise, and manufacturing the filament with the required diameter might not have been possible. The next step relied upon sample printing. Of course, pure PLA samples were printed at the recommended temperature (225–230 °C), but in the case of PLA with graphene pellets, the temperature was adapted in order to obtain the correct and continuous structure. Therefore, after analysing and testing, the temperature of printing PLA-graphene samples ranged from 205–220 °C. This is related to the selection of an appropriate processing temperature of the biothermoplastic. From the point of view of the quality of products made of thermoplastic biopolymers, it is most advantageous to use the lowest processing temperature. With the increase in the processing temperature of PLA (biopolymer), the rate of thermal degradation of the material (not proportionally but exponentially) increases. This applies to any bioplastic. Another important factor influencing the quality of the product is the time that the bioplastic is kept at a specific temperature. Pure PLA was printed at the lowest possible temperature allowed by the manufacturing technology used (selection of parameters based on flow and the efficiency of the nozzle for movement on the work table). This was to minimize the occurrence of the thermal degradation process. 

**Figure 2:**
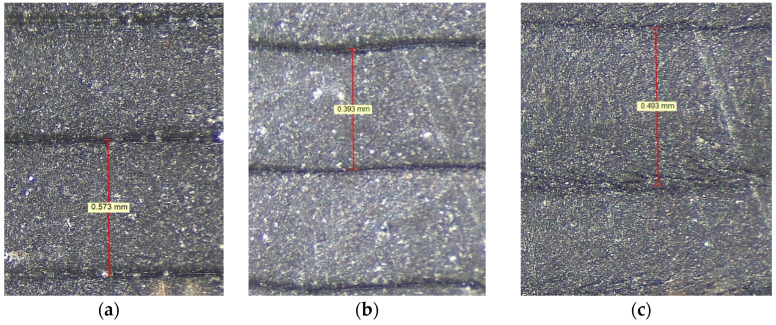
Microscopic views of PLA samples with: (**a**) 1% GNP, (**b**) 2% GNP and (**c**) 4% GNP.

The addition of graphene decreased (slightly but noticeably) the flow of the mixture due to the increase in compounding density (PLA filament/graphene), which resulted in an uneven outflow of polymer from the printing nozzle. By increasing the temperature in composite PLA/graphene, the blends decreased in viscosity. In this case, the printing temperature was also set as low as possible to improve and even the outflow of the composite from the nozzle and avoid excessive degradation. Every sample was printed out using the Prusa i3 MK3 printer with a parameter setting of 100% infill. The printing trajectory was set to be 0–90° along the longitudinal axis of samples. The main printing parameters were assumed: velocity 10–70 mm/s and a single printing layer of 0.05 mm. To investigate the structure of samples, microscopic examinations were performed by employing a Leica s9i microscope with a maximum magnification of 16×. Figure 2 shows the exemplary views of a structure for printed samples with 1%, 2% and 4% GNP. As it was observed, the general width of a single printed strip was between 0.3–0.6 mm, but the diameter of the nozzle was 0.7 mm. 

### 2.2. Tensile Test

**Figure 3:**
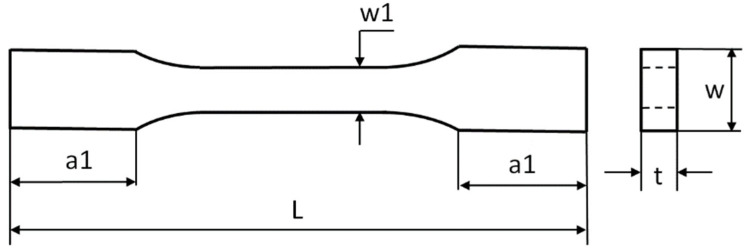
Dimensions of the sample subjected to tests.

The tensile tests on samples were performed using a Shimadzu AS-X testing machine based on standard UNE EN ISO 527-1:2020-01. These tests were carried out with a constant velocity of moveable traverse of 2 mm/min. As a result of stretching the samples, the plots of force vs elongation were obtained. The dimensions of the samples were based on a standard: L = 80 mm, a1 = 15 mm, w1 = 5 mm, w = 10 mm, and mean thickness t = 4 mm (Figure 3). Mean weights of the samples amounted to: 1.6 g (pure PLA) and 1.8–2.1 g (PLA + GNP). Young’s modulus was determined by employing an extensometer with a gauge length of 25 mm. 

### 2.3. Life Durability

One method of determining a material’s life is treating the sample with UV radiation, determining the material’s ageing. The tested samples were placed on an appropriate stand in a glass specially lined with reflective foil. The closed container with the investigated samples was subjected to ultraviolet lamp action with a power of 35 W/cm^2^. It is worth mentioning that the average power of UV radiation obtained on the earth coming from the sun is 62 W/m^2^. This means that one hour of material ageing in an applied container corresponds to about 5645 h of an exposure to a solar radiation. The prepared container was connected to a vacuum pump, forcing airflow. As a result, the ozone formed in the chamber was pumped out. The method was based on loose arrangements of samples on special bases. The entire irradiation process lasted 4 h. A four-hour exposure is equivalent to approximately one year of exposure to solar radiation [18].

### 2.4. Difference Termogravimetric (TG)

With the aim of thermal stability determination, thermogravimetric analysis (TGA) and DTG analyses were used. Thermogravimetric analyser-TGA 550 Discovery of TA Instruments were applied for measures. The tests were conducted in an atmosphere of nitrogen in the scope of temperature from 40 °C to 600 °C with a heating velocity of 10 °C/min.

### 2.5. Dynamic Mechanical Analysis (DMA)

DMA (DMA 850 Discovery of TA Instruments) was used by mounting the sample on the device, but the middle part of the sample was subjected to oscillating motion with a frequency of 10 Hz and amplitude of 10 µm. Firstly, the sample was cooled down to −60 °C and subsequently was heated up with a velocity of 5 °C/min to a temperature of 80 °C.

### 2.6. Differential Scanning Calorimetry (DSC)

DSC tests were performed by means of scanning the differential calorimeter from the Mettler Toledo company. For controlling and data elaborations, STARe Thermal software was applied. The tests were executed in the nitrogen atmosphere at a flow of 30 mL/min.

## 3. Results

### 3.1. Tensile Test

**Figure 4:**
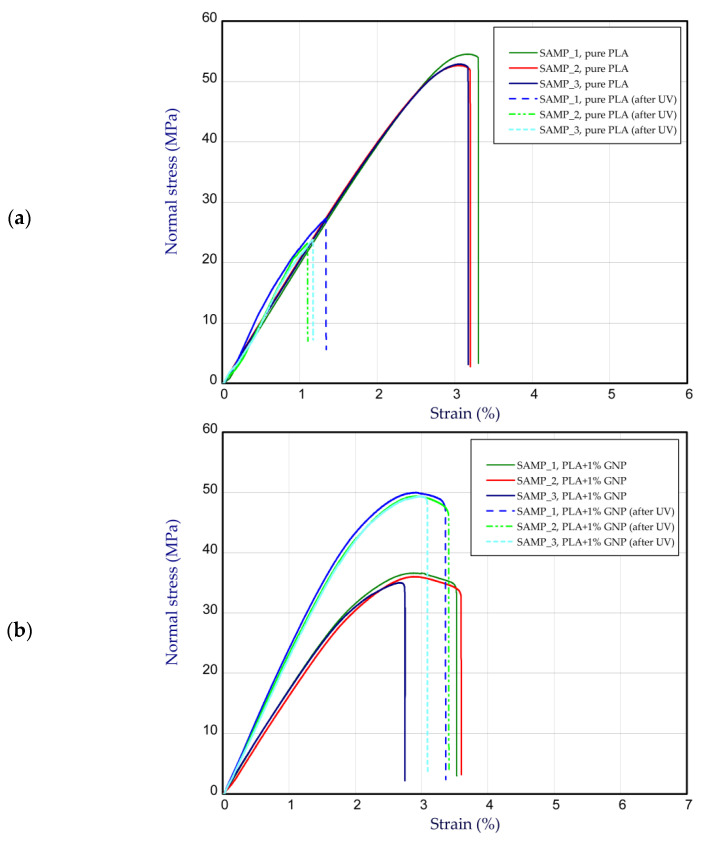
The curves of tensile tests (**a**) of pure PLA and (**b**) PLA at wt. 1% GNP.

**Figure 5:**
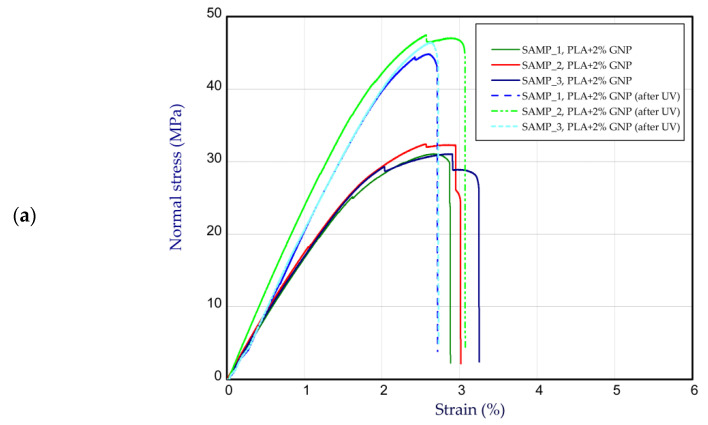

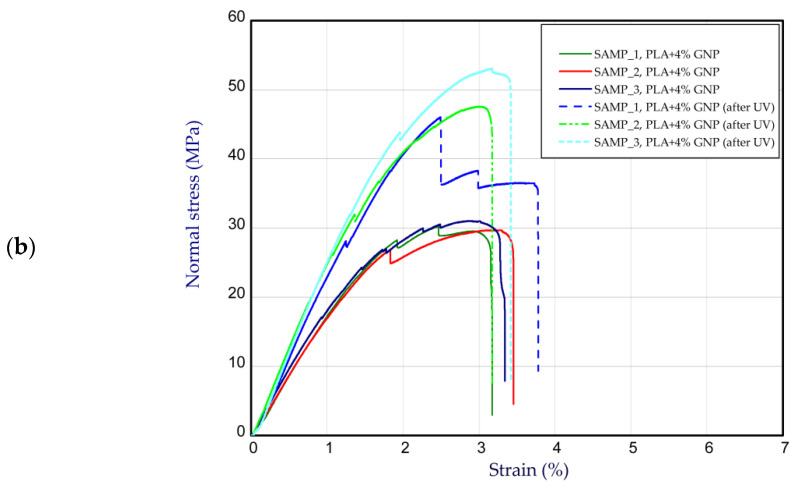
The curves of tensile tests of PLA (**a**) at wt. 2% and (**b**) at wt. 4% GNP.

This subsection presents the results of the tension of considered samples before and after UV treatment. Figure 4 illustrates the curves of normal stress vs strain for pure PLA (Figure 4a) and PLA with the additive of GNP (Figure 4b). In general, the average maximum stress of pure PLA samples before and after UV treatment amounted to above 51 MPa and 18 MPa, respectively. This is a drop of about 3 times. In the case of the stiffness parameter, the mean Young’s modulus was found to be 3.46 GPa and 2.61 GPa (after UV treatment). The situation was reversed when considering PLA + 1% GNP samples (Figure 4b). This means that, in general, the presence of graphene in PLA weakens the strength (33.4 MPa), but after UV radiation, the strength (ultimate stress) increased to 46.6 MPa. In the case of Young’s modulus, mean values were registered at 2.91 GPa and 3.78 GPa. Thus, a content of 1% GNP can raise the stiffness slightly after ageing. Analysing the samples with wt. 2% and 4% GNP, the trend is similar (Figure 5). Firstly, the increase in GNP in samples does not allow for increasing the maximum stress. Secondly, the discrepancy in strength and stiffness between PLA wt. 2% GNP samples (Figure 5a) and PLA wt. 4% GNP samples (Figure 5b) is not great, but it was observed that in the case of the latter, the mean Young’s modulus was the greatest of all considered samples (4.14 GPa after UV treatment). The average values are set out in Table 1. It should be stated that 3D printing is a specific method of producing thermoplastic materials. After printing, the finished element is made of layers (because each layer is applied to the next one, where the previously applied one has already passed into a solid from the liquid phase). As a result, the crystalline structure of the polymer is layered. This means that on the edge of the layer, there is an amorphous phase bonded to the amorphous phase of the next layer, and in the middle of each layer there is a crystalline phase. It is also worth mentioning that 3D printing is related to the so-called print resolution, i.e., the compaction of the material in the working area of the overtaken element/sample.

The application of UV rays caused the supply of high-energy radiation to the PLA crystal lattice, which resulted in the shortening of the main PLA polymer lattice (degradation by electromagnetic radiation). Shorter chains are easier to change position in relation to other chains that build the polymer structure (lower coefficient of viscous friction). Shorter chains and the current energy available in the system (from UV radiation) caused the possibility of PLA crystallization. This situation meant that after UV, an increase in PLA strength was noted; however, this is a negative situation in the long term, as DMA tests proved that the storage module for non-aged samples was higher in the entire range of temperature. Static tests showed only the resistance of the crystal lattice at one temperature (room temperature), with the possibility of easy lattice relaxation (low test speed). There were no chain movements, and the main factor determining high strength was the quality and quantity of the crystal phase in the 3D print. The changes in the network were also indicated by a large dispersion in the static test of prints after ageing.

Based on the data obtained from mechanical tests, it can be noticed that the statistical dispersion of the obtained results increases with the amount of graphene in PLA. This may indicate that the dispersion of the nanoclave in the PLA matrix deteriorated despite the use of solvent dispersion in DCM prior to granulation.

### 3.2. TG Results

**Figure 6:**
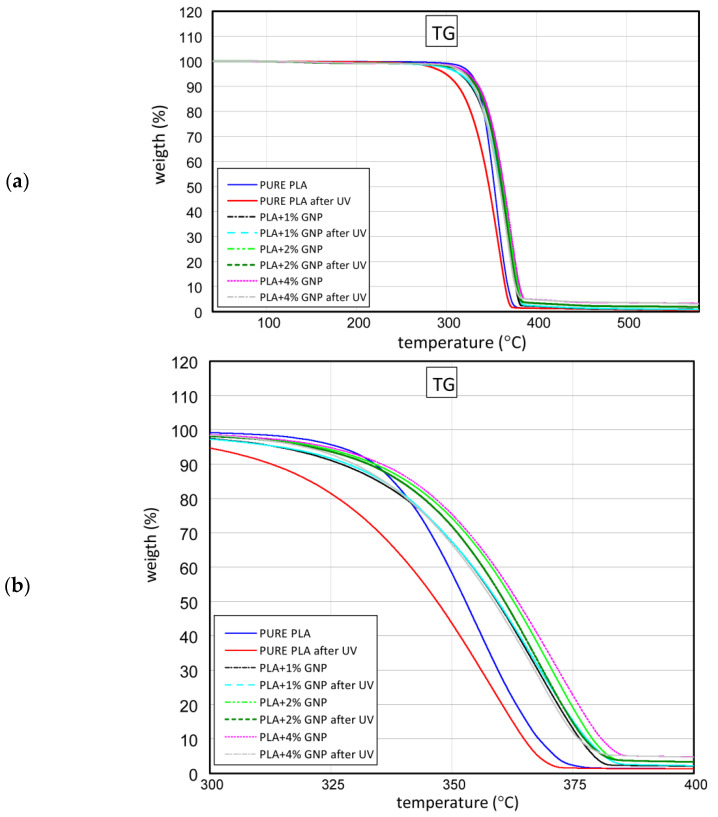
The curves of TG test in the fully realised range (**a**) and in range of 300–400 °C (**b**).

This subsection presents the results of TGA. Figure 6a shows the curves of the weight loss obtained at temperatures of 40 °C to 600 °C, but to see discrepancies better, the diagram has been scaled in the range 300–400 °C (Figure 6b). After tests, it is seen that pure PLA samples after UV radiation have lower thermal stability. It is worth mentioning that the presence of graphene in samples increases thermal stability. The content of graphene at just 1% positively influences the growth of weight loss. The difference between samples with the graphene additive before and after the UV process is insignificant. By increasing the graphene in samples, the thermal stability increases as well. The data obtained by the TG test are summarized in Table 2.

Taking into account that the greatest differences in the rate of weight loss were recorded in pure PLA samples compared to pure PLA after UV radiation. The reason for this is primarily the translucency of the PLA transparent radiation and the penetration of high-energy UV radiation of the PLA chains’ presence in the depths of the sample. The degradation and shortening of the polymer chains are the fastest in this case, because degradation takes place throughout the volume of PLA. Graphene retains and disperses high energy from radiation, and in addition blocks UV rays from penetrating into the PLA/graphene biocomposite. With the increase in graphene content, the thermal stability slightly increases (more UV radiation is absorbed and/or reflected, which provides protection for the PLA chains against radiation).

### 3.3. DMA Results

**Figure 7:**
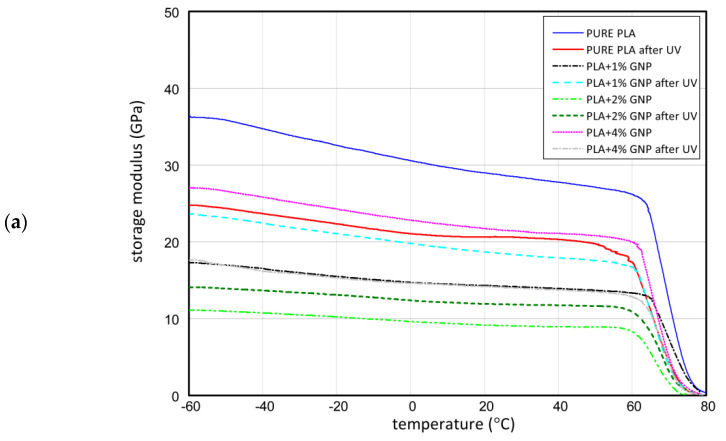

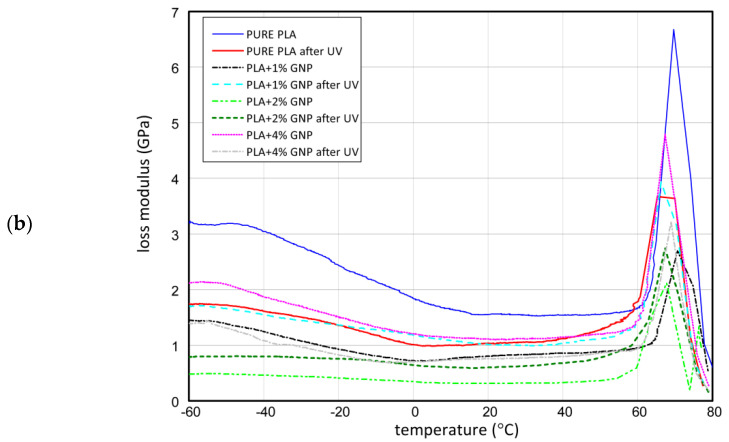
The curves of storage modulus (**a**) and loss modulus (**b**) in range of −60 °C up to 80 °C.

Based on the results of DMA, graphene applied in printed samples decreases the value of behaviour modulus (Figure 7a,b). This is caused by the fact that layers of graphene distributed between PLA chains simplify the slip of those chains and increase the distance between them, weakening their bonds with each other. The obtained results for the behaviour modulus show that this parameter depends on the applied graphene modifier. The lowest drop of behaviour modulus was observed for samples with a graphene content of 4%. This means that the lattice of PLA with this content is the most crystal (graphene becomes a nucleator during crystallisation). The greatest decrease in behaviour modulus was noticed for the sample with 2% graphene. This means that in the range of 0 to 4% graphene, one can distinguish different types of materials (different inner structures of PLA chains). The exciting relationship that was observed is the growth of loss modulus after the UV process for samples with 2% graphene. In this case, the UV radiation caused relaxation of the structure of PLA chains and apparent strengthening of the material (higher behavioural elasticity). A similar phenomenon was observed in the composite with 2% graphene; nevertheless, the effect was weaker in this case. 

The samples containing 4% graphene lost the value of a loss modulus after the ageing process. This can be explained by the fact that if the content of graphene in the PLA sample is too great, and then the graphene might have become a simple insertion. The loss moduli were also subjected to change under the influence of graphene presence and the ageing process. The single graphene decreased a loss modulus, meaning that in composite, more crystal phase exists than in the structure of a pure PLA sample. The UV process hydrolysed the PLA chains; therefore, the loss modulus was lower after the ageing process. In the case of modulus loss, composites with 1% and 2% graphene were characterised slightly by greater values of loss moduli after the UV process. It transpired that shorter chains in the presence of 1% or 2% graphene could easily move to each other at small distances. The characteristic values of DMA are shown in Table 3.

DMA studies on modified (plasticized) PLA are known in the literature [27,28]. In the case of testing pure PLA and modified PLA, similar results were obtained. As the modifier increased, the storage module decreased and the TG temperature decreased by a few degrees with the modifier [24,27]. DMA tests were executed for pure PLA and PLA with graphene, aged in a special chamber (UV, air flow and moisture), which intensified the degradation processes. In the case of pure PLA ageing, the degradation process takes place very slowly, while the temperature at which PLA is aged, is very important [29]. The influence of graphene on the ageing process of the printed PLA matrix and the description of this process using the obtained DMA results in the literature has not been studied so far.

### 3.4. DSC Results

**Figure 8:**
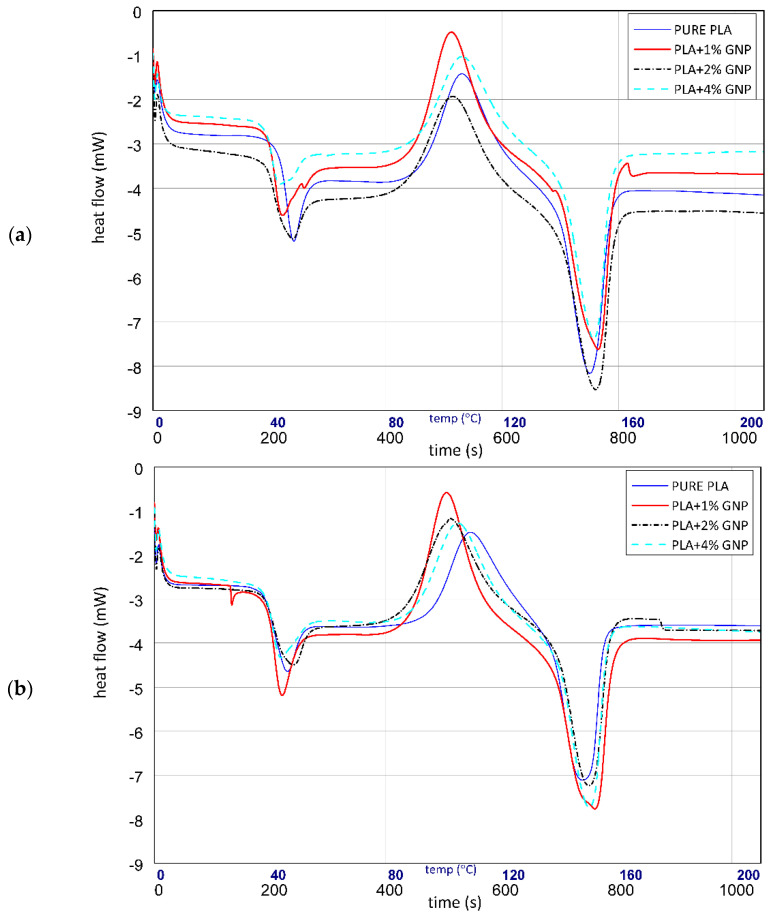
The curves of DSC on samples before (**a**) and after (**b**) UV ageing.

The DSC study of the produced composites allowed us to determine the changes in the structure of the PLA polymer under the influence of UV radiation, under the influence of the addition of graphene and its amount in PLA (ii), under the influence of graphene content on changes in the structure of the PLA/graphene composite, and the influence of UV radiation (iii). Figure 8 shows the DSC curves of PLA samples and PLA/graphene composites (Figure 8a) and PLA samples and PLA/graphene composites after UV ageing (Figure 8b).

Graphene has a significant influence on the structure in which PLA crystallises. The research shows that the best amount of graphene tested in PLA is 1%. This significantly increased the energy of cold crystallisation (10% ↑). This means that graphene acts as a nucleation centre to accelerate PLA to form a crystalline phase. With the increase in the amount of graphene in PLA, the value of cold crystallisation energy decreased, which proves that above 1% graphene, the nanoadditive forms aggregate or there is too much of it between PLA chains, making it difficult to crystallise the polymer chains (cold crystallisation energy value for the graphene content of 4% is almost the same level as in the case of pure PLA). The presence of graphene in the PLA matrix delayed the effects of degradation under the influence of UV light. Exposing pure PLA to UV action caused a slight decrease in the energy of cold crystallisation and a slight increase at the beginning of cold crystallisation. Adding 1% graphene to PLA increased the energy of cold crystallisation by 10%. This amount of additive made the ability to form PLA crystals under the influence of graphene the most effective even after degradation. The addition of 4% graphene in PLA is characterised by similar values of cold crystallisation energy to the raw PLA samples not subjected to UV treatment. With the increase in graphene content, the glass transition temperature and the energy of this transformation of the tested printed PLA polymer decreased. With 1% graphene, the decrease in the energy and temperature of this transformation was the most noticeable—by as much as 4 °C in the case of temperature and the decrease in energy value by as much as 0.1 J/gK (decrease of 20%). The glass transition temperature drop for PLA samples with 2% and 4% graphene was at the level of 5 °C and 6 °C, compared to the glass transition temperature of pure PLA. The energy value of this transformation was maintained at the level of the energy value of the composite containing 1% graphene; interestingly, the decrease in the phase transition energy of the glass transition was at the level of composites containing 1% PLA. The decrease in temperature (and thus the reduced amount of energy) of the glass transition, as well as the value of the energy of this phase transition, was caused by the increase in the distance between PLA chains due to the presence of 2D graphene. Graphene allows the polymer chains to slide between each other because it allows the chains to slide on their surface, and the increase in the interchain distance meant that the phase changes associated with the glass transition required lower energy values. Exposing pure PLA to UV action caused a decrease in the glass transition start temperature. The data obtained from the DSC test are summarized in Table 4.

The addition of graphene stopped this temperature drop; the strongest effect was in the case of 1% graphene in the PLA matrix, and the sample containing 4% graphene had almost identical glass transition parameters to the pure PLA sample after UV treatment. In the case of the scattering of UV radiation on the carbon nanoadditive, one would obtain an increase in UV resistance along with an increase in the amount of graphene in the composite with an increase in its amount. There was a decrease in parameters (mainly Tg temperature values), which means that the enhancement of the UV resistance by graphene is on a structural basis, and its too-high presence in the PLA matrix may lead to a decrease in this resistance compared to pure PLA. The phase transition energy, which is melting from the solid phase to the liquid phase of PLA, was at a similar level for PLA, PLA 2% and PLA 4% graphene. The melting energy for the PLA 1% composite was 20% higher, which proves that in these samples, graphene acted as a nucleation centre, facilitating the formation of PLA crystal structures. The temperature of the beginning of the PLA melting transformation was similar for all samples. The addition of graphene in the PLA matrix did not change the nature of the PLA melting transformation in the composite. The fusion energy values and the temperature range over which this transformation occurred were similar. This means that the degree of degradation of PLA and the hydrolysis of the biopolymer chains under the influence of light was not great because graphene causes the non-transparency of the PLA/graphene composite, and thus the degradation progress is lower, as it takes place only on the surface and propagates deep into composite much slower than in the case of raw light-transparent PLA samples).

## 4. Conclusions and Final Remarks

This paper presents the results of mechanical and physical-chemical parameters of printed pure PLA and PLA admixed with GNP. We determined the influence of graphene on the mechanical properties and the modulus of elasticity. In our research, we investigated the loss or improvement of the properties of biocomposites as a function of temperature and time after exposure to UV radiation in the ageing chamber. Based on the DSC and TG studies, the changes taking place in the structure of the biopolymer were determined. This allowed us to determine the real impact of graphene on the PLA matrix, which we have looked at comprehensively: graphene does not have significant influence on mechanical properties; therefore, graphene should not be added as modificator. We also described the degradation process of PLA in the presence of graphene. The essential aspects of graphene addition can be stated as:

- The presence of GNP in PLA samples caused a slight decrease in Young’s moduli before UV radiation. However, after UV ageing, this parameter was greater compared to pure PLA (before radiation). Of course, after UV treatment, pure PLA was characterised by a weaker Young’s modulus and maximum stress. The content of GNP 1%, 2%, and 4% in samples indicated comparable strength;
- DMA results on GNP samples showed a decrease in storage modulus. The lowest drop of storage modulus was observed for 4% GNP samples. The greatest decrease in storage modulus was noticed for the sample with 2% graphene. It is seen that the loss modulus of aged samples containing 4% GNP decreased;
- TG results proved that the presence of GNP in samples enhanced thermal stability. The content of graphene of at least 1% positively affects the growth of weight loss.
- Graphene has a significant influence on the structure in which PLA crystallises. This is due to its direct influence on the formed crystal structure of the polymer. With the increase in the amount of graphene in PLA, the value of the cold crystallisation energy decreases, which proves that graphene can form aggregates;
- With the increase in the content of graphene, the glass transition temperature and the energy of this transformation of the tested printed PLA polymer decreases, and the exposure of pure PLA to UV action causes a decrease the temperature of the glass transition at the beginning;
- The presence of graphene in the PLA matrix delays the effects of degradation under the influence of UV light. The addition of graphene does not change the nature of the PLA melting transformation in the composite. This means that the degree of PLA degradation and the hydrolysis of the biopolymer chains under the influence of light is not great and is related to the transparency of the printed composite;
- Graphene changes the rheology of the bioplastic filament and requires a higher pre-production temperature. It also dissipates UV energy in the composite and protects PLA chains against radiation penetration into the composite.

## Figures and Tables

**Table 1:** Characteristic parameters obtained in tensile tests for all samples.

Sample	Mean Young’s Modulus E (GPa)	Mean Maximum Stress R_m_ (MPa)	Mean Strain at Maximum Load (No Unit)
Pure PLA	3.46 ± 0.03	50.5 ± 3.1	3.08 ± 0.03
Pure PLA (after UV)	2.61 ± 0.04	18.0 ± 2.4	1.20 ± 0.07
PLA + 1% GNP	2.91 ± 0.02	33.3 ± 2.2	2.81 ± 0.04
PLA + 1% GNP (after UV)	3.78 ± 0.04	46.6 ± 4.3	2.64 ± 0.09
PLA + 2% GNP	2.88 ± 0.02	29.5 ± 2.5	2.80 ± 0.11
PLA + 2% GNP (after UV)	3.76 ± 0.06	42.2 ± 4.6	2.56 ± 0.17
PLA + 4% GNP	3.01 ± 0.03	27.8 ± 3.4	3.02 ± 0.11
PLA + 4% GNP (after UV)	4.14 ± 0.06	44.5 ± 6.4	2.82 ± 0.26

**Table 2:** Weight loss for PLA samples and PLA/graphene composites.

Sample	T_5%_ (°C)	T_10%_ (°C)	T_onset_ (°C)	T_endset_ (°C)	DTG_max_ (°C)	m_loss_ (%)
Pure PLA	327	334	332	370	358	98.738
Pure PLA (after UV)	299	313	312	368	359	98.457
PLA + 1% GNP	314	327	330	380	368	97.720
PLA + 1% GNP (after UV)	315	329	331	367	381	97.349
PLA + 2% GNP	323	334	334	381	370	97.125
PLA + 2% GNP (after UV)	321	333	333	379	358	96.626
PLA + 4% GNP	324	336	337	382	373	95.350
PLA + 4% GNP (after UV)	320	330	330	377	369	95.465

T_5%,10%_—temperature at which 5, 10 percent of the mass is lost. T_onset, endset_—temperature at the beginning and end of the degradation. DTG_max_—temperature at which the degradation process takes place most intensively. M_loss_—weight loss at 400 °C.

**Table 3:** Characteristic parameters read from charts based on DMA.

Sample	E’Change (GPa)	E’Onset (°C)	E’’Onset (°C)	E’’Peak x (°C)	tanδpeak x (°C)
Pure PLA	12.9	63.5	−29.8	69.6	77.8
Pure PLA (after UV)	7.12	58.8	−29.2	69.9	74.1
PLA + 1% GNP	4.38	64.7	−44.5	70.7	78.8
PLA + 1% GNP (after UV)	7.64	60.6	−47.5	66.3	74.9
PLA + 2% GNP	2.44	59.3	−31.5	67.7	71.8
PLA + 2% GNP (after UV)	2.67	57.8	−21.2	67.4	75.9
PLA + 4% GNP	8.67	61.5	−46.3	67.3	75.9
PLA + 4% GNP (after UV)	5.19	62.4	−47.9	69.0	74.2

E’ change—decrease in the storage modulus from—60 degrees to the beginning of the glass transition (E’onset). E’onset—the beginning of the glass transition is associated with a very sharp decrease in the storage module. E’’peak x—the beginning of the increase in the value of the loss modulus. tanδpeak x—the temperature at which the ratio of the loss modulus to the conservative modulus (E’’/E’) is the highest.

**Table 4:** The data obtained by the DSC for PLA samples and PLA/graphene composites.

Sample	Tg_midpointISO_ (°C)	Tg_∆CpISO_ (J/gK)	T_kr_ (°C)	Q_kr_ (J/g)	T_m_ (°C)	Q_m_ (J/g)
Pure PLA	61.1	0.530	113	22.58	150	23.76
Pure PLA (after UV)	57.6	0.494	116	21.08	148	20.72
PLA + 1% GNP	58.4	0.434	110	24.94	152	22.29
PLA + 1% GNP (after UV)	57.6	0.546	109	24.38	151	26.33
PLA + 2% GNP	58.7	0.477	111	23.61	151	23.48
PLA + 2% GNP (after UV)	58.1	0.467	110	23.78	151	22.45
PLA + 4% GNP	57.9	0.470	113	22,53	152	23.52
PLA + 4% GNP (after UV)	57.5	0.385	112	22.07	151	23.64

Tg_midpointISO_—glass transition temperature. Tg_∆CpISO_—energy of transformation of the glass transition. T_kr_—temperature at which cold crystallization takes place most intensively (maximum peak). Q_kr_—cold crystallization Energy. T_m_—melting point (maximum peak). Q_m_—melting point energy.

## Data Availability

Not applicable.
